# Auto-Refining Reconstruction Algorithm for Recreation of Limited Angle Humanoid Depth Data

**DOI:** 10.3390/s21113702

**Published:** 2021-05-26

**Authors:** Audrius Kulikajevas, Rytis Maskeliūnas, Robertas Damaševičius, Marta Wlodarczyk-Sielicka

**Affiliations:** 1 Department of Multimedia Engineering, Kaunas University of Technology, 51368 Kaunas, Lithuania; audrius.kulikajevas@ktu.lt (A.K.); rytis.maskeliunas@ktu.lt (R.M.); 2 Faculty of Applied Mathematics, Silesian University of Technology, 44-100 Gliwice, Poland; robertas.damasevicius@polsl.pl; 3 Department of Geoinformatics, Maritime University of Szczecin, Waly Chrobrego 1-2, 70-500 Szczecin, Poland

**Keywords:** pointcloud reconstruction, adversarial auto-refinement, human shape reconstruction

## Abstract

With the majority of research, in relation to 3D object reconstruction, focusing on single static synthetic object reconstruction, there is a need for a method capable of reconstructing morphing objects in dynamic scenes without external influence. However, such research requires a time-consuming creation of real world object ground truths. To solve this, we propose a novel three-staged deep adversarial neural network architecture capable of denoising and refining real-world depth sensor input for full human body posture reconstruction. The proposed network has achieved *Earth Mover* and *Chamfer* distances of 0.059 and 0.079 on synthetic datasets, respectively, which indicates on-par experimental results with other approaches, in addition to the ability of reconstructing from maskless real world depth frames. Additional visual inspection to the reconstructed pointclouds has shown that the suggested approach manages to deal with the majority of the real world depth sensor noise, with the exception of large deformities to the depth field.

## 1. Introduction

One of the most rapidly expanding scientific research fields, thanks in part to the advancements in artificial intelligence and, specifically, Deep Neural Networks (DNNs), is computer vision. Whereas regular cameras have already been widely adopted in various object detection tasks, however, depth sensors still have narrow range of research dedicated to them. This can be attributed to them not being easily available for personal use until relatively recently with the introduction of the original *Kinect* sensor [1]. Unfortunately, while the *Kinect* technology made the depth sensors affordable they have not had wide consumer adoption outside of entertainment [2,3], although health-related applications were also considered [4,5,6]. This is, however, likely a rapidly shifting trend with more consumer grade sensors, such as *Intel Realsense* [7], being released and depth scanning systems being integrated as part of mobile devices. With rapidly evolving field of three-dimensional object reconstruction, such depth sensing systems [8,9] may be the key to boosting object reconstruction quality.

Quite a few real world applications would benefit greatly from depth sensors and/or real-time object reconstruction, starting with collision avoidance in autonomous vehicles [10,11], robotics [12,13], or posture recognition [14,15]. Other object reconstruction applications may involve interactive medium, like obstacle avoidance in virtual reality [16,17], augmented reality [18], extended reality [19], and more. Even though 3D object reconstruction opens up a lot of possibilities to various fields, the main issue with object reconstruction is that it generally requires either intricate camera setups or moving the camera around the object in order to scan the entirety of the object and to build its full profile from all sides. This makes the reconstruction have a high accessibility barrier, as daily users cannot be expected to have professional filming setups containing laser sensors arrays capable of single-shot scanning entire object from all perspectives, nor expected to bother carefully scanning an object from all of its sides to reconstruct the object iteratively. This is in conjunction with potentially requiring a lot of computing power to perform high fidelity pointcloud fusion, which greatly impacts end-user experience.

Therefore, there is a strong desire for a solution that is able of performing object reconstruction task by using only a single camera view. There already are existing solutions that attempt to solve the previously mentioned problems with multi-view perspective reconstruction by using a priori knowledge. These methods usually involve using artificial intelligence, specifically deep neural networks which are able to approximate the occluded object information leveraging reinforcement learning. This type of learning is reminiscent of how a person is able to generally infer the objects shape based on the mental object model they had built from their life experience. There already exist methods capable of performing object reconstruction from a single perspective, one of the most popular solutions due to its simple implementation is volumetric pixel (voxel)-based reconstruction. However, high fidelity models using voxel-based models require large amounts of operative memory to represent.

To mitigate this, certain reconstruction solutions instead of attempting to reconstruct voxels try to predict pointclouds. Unlike voxel-based solutions, pointclouds require very little memory overhead for their representation. However, their comparison functions are much more complex, making them a lot harder to train due to vertices being able to occupy any coordinate in three-dimensional space. One of the first of such type of solutions being *PointOutnet* [20], which has shown the capability of predicting and generating plausible 3D shapes of objects. Although the solution has shown good prediction results, it relies on hand drawn object segmentation masks, which makes this solution not really applicable to real world applications. Additionally, this solution worked on flat 2D images, which lose a lot of important depth information. Nevertheless, there are already existing solutions capable of leveraging pointcloud information in order to improve generalization and prediction quality [21]. However, these solutions generally are only applicable to either synthetic or manually pre-processed real world data, which makes them unsuitable for real-time applications.

To solve this, we propose a novel unsupervised adversarial auto-refiner capable of full human body pointcloud reconstruction using only a single self-occluding depth view capable of reconstructing real depth sensor data with no masking nor any other direct interference. Our contribution to the field of reconstruction is the three-stage (cleanup, course, and fine) adversarial network capable of cleaning up the noise from real world input, without losing the underlying shape or position of the body posture.

The structure of the remaining parts of the paper is as follows. Section 2 discusses the related work. Section 3 describes our synthetic dataset and the proposed methodology. Section 4 presents the results. Section 5 discusses the results of this study. Section 6 presents the conclusions.

## 2. Related Work

Thanks to advancements in artificial intelligence and deep neural networks, object reconstruction is a rapidly expanding computer vision field. Currently, there are two main approaches in order to reconstruct a 3D, voxel-based and pointcloud-based. One of the most well known voxel-based solutions is *3D-R2N2* [22] that uses *Sanford Online Products* [23] and *ShapeNet* [24] datasets as a priori knowledge in order to predict objects shape using both either single or multi-perspective reconstruction. It uses deep recurrent neural networks with *Long Short Term Memory* [25,26] (LSTM) to learn objects features using multiple views as training input, while still being capable of predicting objects shape from a single perspective when performing predictions. Unfortunately, the method requires additional masks that need to be provided separately in order to make a prediction. One of the solutions that attempted to resolve this drawback has extended *YoloV3* [27] network by merging the reconstruction and object prediction tasks (*YoloExt*) [28]. Unlike *3D-R2N2*, it was capable of detecting and performing *Region of Interest* (RoI) segmentation independently before passing the mask to the object reconstruction branches. This solution allowed it to be independent of outside interference and could work with real world input. Some other attempts were made using hybridized neural network models [29] which are separately trained on groups of objects for faster model convergence and ability to reconstruct in real-time due to reduced network complexity. Despite voxel-based approaches being easy to represent and train due to low mathematical complexity of loss function, they suffer from a major flaw: the exponential memory requirements in order to train high granularity model, which would be required in order to reconstruct complex models containing a lot of details. While there have been attempts to solve this issue of ever-increasing memory requirements by using more compact data representation styles, such as octrees [30,31], thus greatly reducing the amount of required data to represent the same model, these still suffer from overheads.

However, a much better solution of representing 3D volumes than voxels is pointclouds. Unlike voxel-based solution, pointclouds have much lower memory footprint both during training and prediction stages, this allows for much higher fidelity reconstruction to be performed. Unfortunately, due to their very nature training pointclouds is difficult, due to the high complexity of loss function that is required to compare ground truth and prediction. One of the first solutions being *PointOutNet*. Same as with *3D-R2N2*, it requires an external mask to be provided with the input in order for the network to properly reconstruct from an RGB image. However, unlike its voxel-based predecessor, it is able to reconstruct the shape using unstructured pointcloud. The approach suggests both *Chamfer* [32] and *Earth Mover’s* [33] distances (CD and EMD, respectively) as loss metrics. Subsequent research in pointcloud reconstruction instead of using RGB frame that loses depth information attempted to use pointclouds as inputs [34,35]. One of the main drawbacks when using unstructured pointclouds is that it is not possible to use well-known feature extracting convolutional neural networks, as due to unstructured nature of the pointcloud data both 2D and 3D convolutional kernels are not applicable to the input. To resolve this issue, *PointNet* attempts in learning symmetric functions and local features. When *PointNet* is matched with a fully-connected auto-encoder branch, it was able to fill in missing chunks in malformed pointclouds. Other research proposes the addition of a fine-grained pointcloud completion method; this way PCN [36] manages to maintain only a few parameters during training due to its course-to-fine approach. However, *AtlasNet* [37] suggests the addition of patch-based reconstruction that is capable of mapping 2D information into parametric 3D object groups, while others generally focus on *Chamfer* distance as a loss metric and only use *Earth Mover’s* distance as an accuracy metric. Moreover, EMD is less sensitive to density distribution, and it also has high computational complexity of $O(n^{2})$ for its calculation. An EMD approximation [38], in addition to evenly distributed point sub-sampling method, is proposed for application in *MSN*, which has shown state-of-the-art reconstruction performance.

Table 1 compares different reconstruction solution implementations. As we can see, our solution is capable of performing sensor-to-screen prediction; due to the use of EMD as loss function, we are also able to maintain sensitivity to high density distributions.

## 3. Materials and Methods

### 3.1. Dataset

There are multiple datasets for object detection which contain image data, such as *COCO* [39] and *Pascal VOC* [40], 3D object datasets, such as *ShapeNet*, and even labeled voxel data [41]. Our task requires human meshes that contain ground truth information, in addition to depth camera information which would match positions. Only a few such datasets exist publicly, like *ITOP* [42] and *EVAL* [43]; unfortunately, in both cases, they use *Kinect* sensors, which have been discontinued by *Microsoft*. Thus, any solutions developed for it are obsolete as generally different manufacturer depth sensors have different types depth errors. Therefore, it is up to us to create dataset that matches our specifications. Because creating a dataset that would have real world ground truths is prohibitively time-consuming, as in creating ground truths for each of the frame of a recorded person manually, we have devised two datasets: dataset containing synthetic data and dataset containing real world data. Synthetic dataset contains data frame samples generated using *Blender* [44], while real dataset contains data pre-recorded human poses using two *Intel Realsense* devices.

#### 3.1.1. Synthetic Dataset

**Figure 1:**
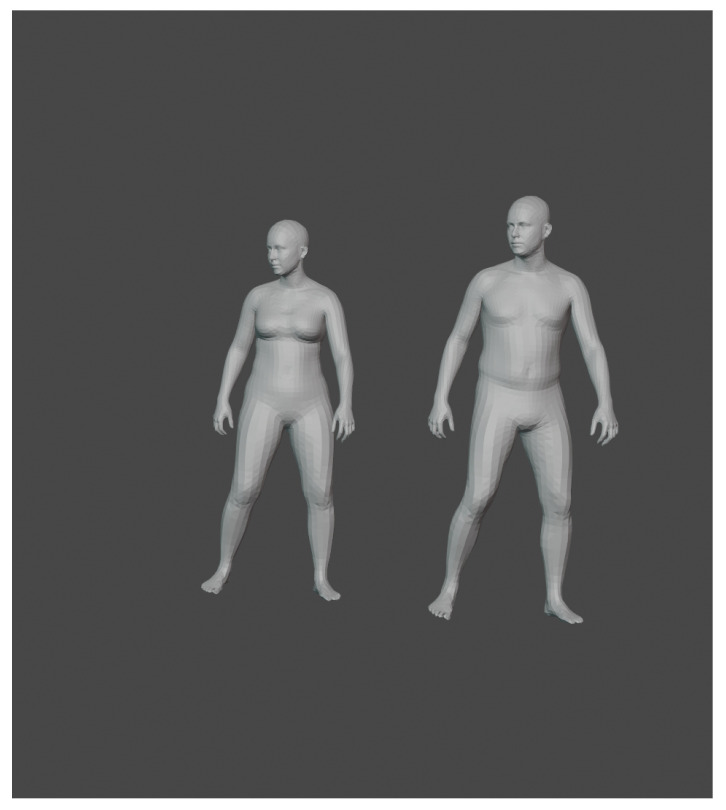
*MoVi* dataset example. Motion capture pose applied to models provided by *AMASS*. Same pose is applied to female and male body type.

To create synthetic dataset, we use *MoVi* [45] dataset as a base as it contains a large library containing motion capture data from multiple camera perspectives. Unfortunately, dataset contains no depth information; to solve this, we bind the motion capture data provided to the *AMASS* [46] triangle meshes. An example of *AMASS* dataset can be seen in Figure 1.

**Figure 2:**
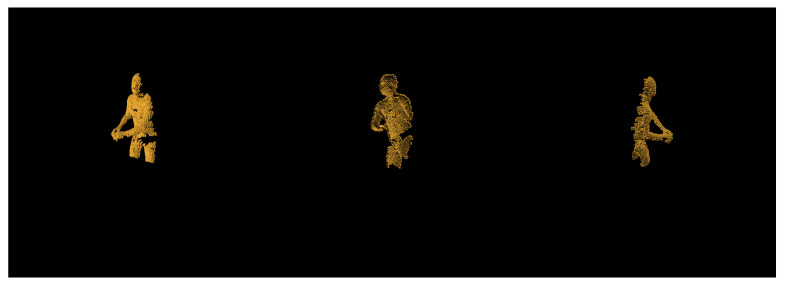
An example of depth frame generated by *Blender* converted into pointcloud using cameras intrinsic parameter matrix *K*.

In order to create the synthetic dataset from the motion captured models, we render the depth maps from various angles by rotating the camera and the human model itself. This is done to create multiple views of the same event from a single motion capture file. The human model is rotated from [0°, 360°) in 45° increments on *Up* (*z*) axis, whereas the camera itself is rotated in ranges of [−35°, 35°] in the increments of 15° on *Up* (*z*) axis. The camera is placed 1.8 m away from person and 1.4 m above ground. This done so that the human position relative to the frame is more in alignment with the real world depth sensor data. The positioned model is then captured using raytracing, and the exported depth frame is saved using *OpenEXR* [47]. This file format is chosen as it does not have any type of compression and is linear and lossless; therefore, it does not lose any depth information that a standard 8-bit channel image format would provide. In addition to the rendered depth frame, we uniformly sample 2048 points of the surface mesh to create ground-truth pointclouds. An example of resampled pointcloud can be seen in Figure 2.

In order to convert a pinhole typed depth camera frame into pointcloud, we use intrinsic parameter matrix K (see Equation (1)), whereby, applying camera intrinsic to each of the pixels in the depth map, we are able to recreate an undistorted pointcloud. The $f_{x}$ and $f_{y}$ denotes the image focal points, while $c_{x}$ and $c_{y}$ is the sensor center point. The intrinsic parameters are applied to the 640×480 depth frame using Algorithm 1, which maps each of the depth pixels to one point in pointcloud. Points with zero depth can be filtered out, while the rest can be resampled using *Farthest Point Sampling* [48] (FPS) to desired resolution pointcloud.

(1) $$K=\left[\begin{array}{ccc} f_{x} & 0 & c_{x}\\ 0 & f_{y} & c_{y}\\ 0 & 0 & 1 \end{array}\right].$$

**Algorithm 1** Convert depth image to pointcloud
1:**procedure**TO_POINTS($w,h,f_{x},f_{y},c_{x},c_{y},D$) ▹ Converts depth D to vertices2:    x←03:    y←04:    V←{∅}               ▹ Create empty output vertex list5:    **for** x < w **do**6:        **for** y < h **do**7:           $z_{i}\leftarrow D\left(x,y\right)$          ▹ Get depth value from depth frame8:           $x_{i}\leftarrow \left(x-c_{x}\right)\cdot z_{i}/f_{x}$9:           $y_{i}\leftarrow \left(y-c_{y}\right)\cdot z_{i}/f_{y}$10:           *insert ($x_{i}$, $y_{i}$, $z_{i}$) into V*11:        **end for**12:    **end for**13:    **return** V14:
**end procedure**



#### 3.1.2. Real World Dataset

**Figure 3:**
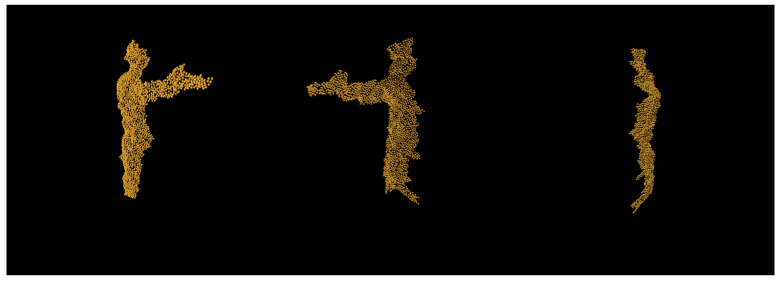
An example of real world depth frame converted into pointcloud using cameras intrinsic parameter matrix *K*.

For our real world dataset, we have captured multiple subjects performing various tasks using two *Intel Realsense* devices. The first depth sensor (*Intel Realsense L515*) is positioned in, while the second depth sensor (*Intel Realsense D435i*) is positioned to 90° side of the subject. The subjects are asked to perform these gestures while being filmed from both angles simultaneously: standing in front camera raise the hand forward, place on top of the head, touch the nose, move the hand to the side, raise the hand above the head, or facing camera with the back raise the hand to the back. No additional preprocessing of the camera depth frames is done outside of cutting off anything further than 2.5 m away from the subject. The depth frame is then converted into pointcloud using each of the sensors intrinsic parameters and resampled using *FPS* to 2048 points. An example of resampled depth frames from each of the devices can be seen below in Figure 3. As we can see, the depth tends to be quite noisy when compared to synthetic, and this makes the existing approaches fail completely or have very poor results when trained on synthetic data.

### 3.2. Proposed Adversarial Auto-Refiner Network Architecture

**Figure 4:**
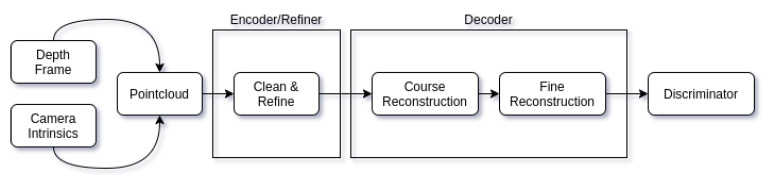
An overview of our adversarial auto-refiner network architecture. It uses captured depth frame and camera intrinsic matrix to convert depth information into a pointcloud. Pointcloud is then fed into encoder stage. Extracted and cleaned up features are sent to decoder where course and fine reconstruction stages take place. The resulting fine reconstruction is then evaluated by the discriminator.

Our proposed adversarial auto-refiner network architecture has three main stages used for object reconstruction. Encoder/Refiner contains the first: cleaning up and refining stage, it is responsible for cleaning out the noise from the original input and capturing the most important features of the pointcloud. The decoder contains two subsequent stages: course reconstruction and fine reconstruction. These three stages are our deliverables and responsible for the pointcloud reconstruction. In addition to these three stages, we also have an additional discriminator network attached at the end of reconstruction that acts as a guide to clean up the noise from the real world depth sensor data and make it *synthetic-like* without losing any of the input features. Overview of the entire network architecture can be seen in Figure 4.

**Figure 5:**
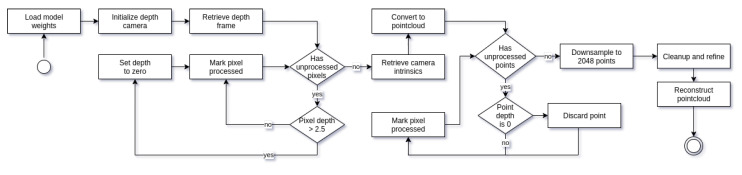
Activity diagram of the proposed method for human posture reconstruction.

Once we have trained our artificial adversarial neural network, we are able to perform sensor-to-screen reconstruction; see Figure 5 for full *UML* activity diagram. To perform a reconstruction, firstly, we initialize the system by loading the trained model weights and initialize the depth sensor; in our case, this is an *Intel Realsense* depth camera sensor. Once the system is ready, we retrieve a single depth frame, as we are not interested in the color information of the captured frame. Afterwards, we filter out invalid pixels by setting all pixels with depth over 2.5 m to zero. This is done to avoid irrelevant background noise. Once we have the filtered depth frame, we convert it to pointcloud using intrinsic camera parameters, and the resulting pointcloud is then filtered again by discarding any of the vertices, in which distance is zero, and flattening the input to a single dimension as the pointcloud itself is not an unstructured data structure. The filtered pointcloud is thereafter downsampled to 2048 points using *FPS* and used as an input in the refiner stage. Refiner outputs input pointcloud features along with cleaned up pointcloud that is then passed through the decoder network for object reconstruction. This gives the final output of reconstructed human mesh with the density of 2048 points.

#### 3.2.1. Refiner

**Figure 6:**
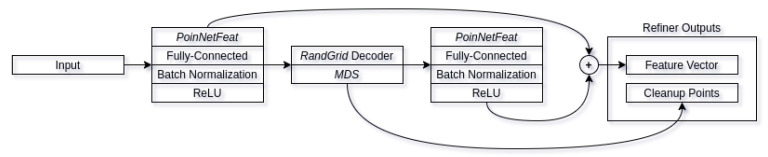
Refiner network architecture. For a given pointcloud, it outputs a cleaned up pointcloud, in addition to a feature vector containing a combination of both cleaned up and original feature vectors.

**Figure 7:**
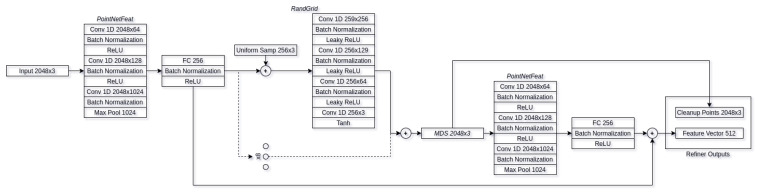
Full refiner network architecture.

The refiner architecture is our main contribution to the field of object reconstruction. While the majority of the applications of adversarial neural networks involve generation of new samples [49,50,51,52], this can be done either from random noise, hand drawn-input, etc. Very little research has focused on refining the initial input without distorting the input. While there have been attempts at refining the synthetic data in order to make it similar to synthetic [53], they still require some sort of knowledge of the given input, as in the case of Reference [53], and it is the pupil direction. Our approach, however, involves of refining real world data to make it more akin to synthetic without knowing any correlation between synthetic and real world data. The refiner network architecture can be seen in Figure 6. Our refiner architecture uses the suggested *PointNetFeat* [21] pointcloud feature extraction architecture directly connected to a fully-connected bottleneck layer of size 256, followed by batch normalization in order to improve generalization and reduce training times [54,55], connected to non-linearity function. For our non-linearity, we use *Rectified Linear units* [56] (ReLU) for they have fast computation times and have been shown to achieve better results when compared to other non-linearity functions, such as *sigmoid*. Output feature vector is then connected a modified random grid (*RandGrid*) decoder architecture, as suggested by Liu et al. [38], using 8 primitives for reconstruction. The output pointcloud is them resampled using *Minimum Density Sampling* (MDS) in order to more evenly distribute the subset pointclouds. Our main modification to the random grid decoder consists of having the uniform distribution for initial random positions be in range of [−0.5,0.5] with the offset of input pointcloud center of mass, in addition to the points being on all three axis instead of two. This has improved the convergence by having the initial points more likely to be distributed around the reconstructed object. Resampled pointcloud is part of the two outputs provided by the refiner/encoder network. It acts as the comparison output for our discriminator network and as part of feature vectors that are used for the decoder. To obtain feature vectors from the refined pointcloud, we apply additional feature extraction block as we did with original input. This gives us two feature vectors of shape 256 that we combine into final feature vector of shape 512 that is then used in the decoders’ reconstruction phases. For full refiner architecture, see Figure 7.

#### 3.2.2. Decoder

**Figure 8:**
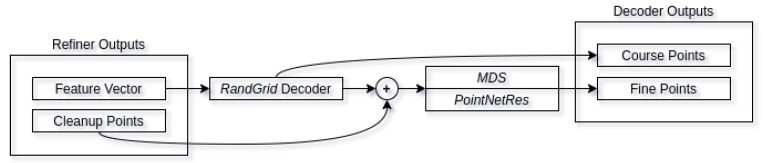
Decoder architecture. Refiner feature vectors are used in order to reconstruct the course human pointcloud. Course features along with cleaned up features are then resampled and passed through residual block, of which output is fine-grained point reconstruction.

**Figure 9:**
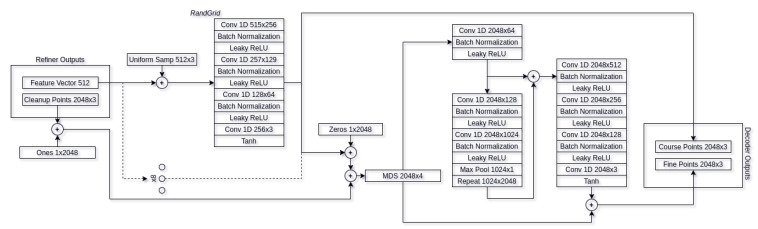
Full decoder network architecture.

Our decoder network resembles Liu et al. [38] decoder architecture, with the main modifications being in the random grid (*RandGrid*) decoder. Unlike the paper suggested architecture, we have modified the random pointcloud to be generated on all three axes, in the range of [−0.5,0.5] with the offset of refined pointcloud center of mass. The overview of the architecture can be seen in Figure 8. The refiner output feature vector is passed to *RandGrid* decoder using 8 primitives for reconstruction, giving the reconstruction of course points. The output course pointcloud is then merged with refined pointcloud instead of the input pointcloud and resampled using minimum density sampling. Resampled pointcloud is then passed through residual decoder (*PointNetRes*) giving the final output of fine pointcloud reconstruction. For full decoder architecture, see Figure 9.

#### 3.2.3. Discriminator

**Figure 10:**
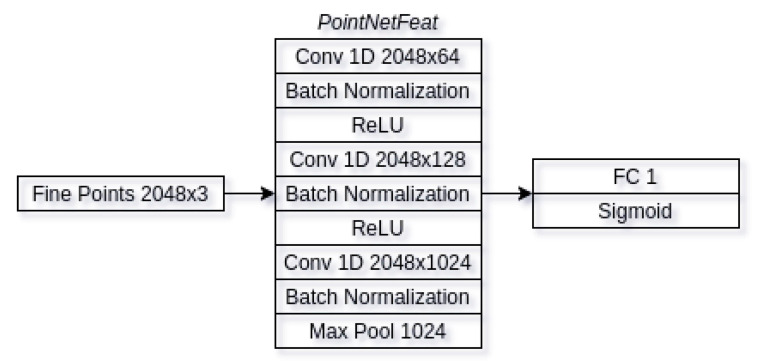
Discriminator architecture. Decoder fine pointcloud reconstruction is used as discriminator input. It is passed through feature extraction block. Extracted features are then connected to fully-connected layer, followed by sigmoidal function.

The main job of discriminator is to evaluate whether given input is either synthetic or real input. Our discriminator is shown in Figure 10 below. We take the output of the decoders fine pointcloud reconstruction and use that as an input in the discriminator. The inputs are passed through feature extraction block (*PointNetFeat*), which is subsequently connected to fully-connected layer. Our fully connected layer only has an output of a single neuron that is then passed through sigmoid function. The output of the sigmoid function indicates if the generated pointcloud is synthetic (1) or if it is real (0). This is done in order to make the input pointcloud as close to synthetic samples as possible as we only have ground-truths for synthetic pointclouds, thus our only being able to train the decoder on synthetic examples.

### 3.3. Training Procedure

To train our neural network, we have chosen a four phase approach: encoder training, decoder training, discriminator training, and refiner training. We have chosen four phased training approach as we have found the network to be much easier to train this way, in addition to having much better prediction results. Additionally, while training, we introduce augmentations to the synthetic input in order to try and produce *Realsense*-like depth deformities. This is done in two ways: either removing random patches from the pointcloud or by adding wavelet disturbances to the pointcloud (see Equation (2)). Wavelets have the period of ω=[2π,32π] and the amplitude of A=(0,0.03]. There is a 75% chance of pointcloud having small patches removed and 50% chance of it having wavelet deformities. These two types of augmentations are expected to be cleaned up during the cleanup stage in the refiner/encoder branch. In addition to these augmentations, there is a third type of augmentation which involves adding random scale factor to the model. This is done because real world people are different heights, while the synthetic models only have single height subjects. Height augmentation also has a 50% chance of applying height scaling in the range of [0.8,1.8].

(2) p(x,y,z)=(x+A·cos(ω·x),y+A·sin(ω·y),z).

#### 3.3.1. Phase I

During first phase, we focus on passing the best weights to the encoder to do this, in this phase we completely ignore the discriminator and ignore its outputs. Instead, we train all three of the reconstruction stages (cleanup, course and fine) to try and act as a regular auto-encoder by passing through input values to output with. To compare the auto-encoder result, we need a loss function capable of comparing unstructured pointcloud data. One of the most popular ones for this task is *Chamfer* distance due to its low memory impact and fast computation. However, what we found is that the use of *Chamfer* produces improper features by causing vertices to congregate close to each other instead of spreading around the desired mesh properly. Therefore, we have instead opted to use *Earth Mover’s* distance (see Equation (3)) along with suggested penalization criteria (see Equation (4)) in Liu et al. [38], where d(u,v) is Euclidean distance between two vertices in three-dimensional space; 𝟙 is the indicator function used to filter which shorter than $\lambda l_{i}$ with λ=1.5 as per suggested value.

(3) $$EMD\left(S,\hat{S}\right)=\underset{\phi :S\to \hat{S}}{min}\frac{1}{\left|S\right|}\sum_{x\in S}||x-{\phi \left(x\right)||}_{2},$$

(4) $$EXP\left(S,\hat{S}\right)=\frac{1}{KN}\sum_{1\leq i\leq K}\sum_{\left(u,v\in \tau_{i}\right)}𝟙\{d\left(u,v\right)\geq \lambda l_{i}\}d\left(u,v\right).$$

During the Phase I training, our final loss function looks like Equation (5), where ${\hat{S}}_{clean}$ is the result of the cleaning stage, ${\hat{S}}_{course}$ is the result of course stage, ${\hat{S}}_{fine}$ is the result of fine point reconstruction, and $S_{clean}$ is ground truth for cleaned pointcloud, as the input pointcloud can have additional noise added to it during augmentation. As per previous research, we kept γ=0.1 for the expansion penalty factor. The network stays in Phase I training until $\epsilon_{\Phi 1}>0.13$; this training value was chosen during experiments as a good value to start training next phase. Once this condition is triggered, Phase II of training starts.

(5) $$\begin{array}{c} \epsilon_{\Phi 1}=EMD\left(S_{clean},{\hat{S}}_{clean}\right)+EMD\left(S_{clean},{\hat{S}}_{course}\right)+EMD\left(S_{clean},{\hat{S}}_{fine}\right)+\\ \gamma \cdot (EXP\left(S_{clean},{\hat{S}}_{clean}\right)+EXP\left(S_{clean},{\hat{S}}_{fine}\right)) \end{array}.$$

#### 3.3.2. Phase II

During this training phase, we focus on training the decoder itself; therefore, during this stage, no modifications to the network weights are applied to the refiner or discriminator branches. In addition, unlike in the previous phase, we only train on synthetic dataset. When Phase II condition is triggered for the first time, we need to apply some weight re-initialization to the network in order to clean up previous training phases potential falls into local minimums. This is done to clear all the decoder weights acquired during Phase I training. To drop the weights, we re-initialize all the decoder weights using Xavier initialization [57] and biases with uniform distribution. In addition, we drop any optimizer state that has been built up to this point for both encoder and decoder optimizers. During Phase II, only decoder weights are being modified in order to build a viable profile for both real and synthetic pointcloud reconstructions during Phase III training. Because only decoder weights are being trained during this phase, our loss equation can be simplified to Equation (6), where $S_{gt}$ is the synthetic ground truth for the synthetic input. Phase II is trained while $\epsilon_{\Phi 2}>0.08$; once loss drops below the threshold, Phase III training starts. Like with Phase I, the threshold value has been chosen experimentally.

(6) $$\epsilon_{\Phi 2}=EMD\left(S_{gt},{\hat{S}}_{course}\right)+EMD\left(S_{gt},{\hat{S}}_{fine}\right)+\gamma \cdot EXP\left(S_{clean},{\hat{S}}_{fine}\right).$$

#### 3.3.3. Phase III

During this phase, we are concerned with training the discriminator to differentiate between real and synthetic input predicted pointclouds. Training discriminator up until this point makes the weights very unstable; for this reason, training it was relegated to its own phase. The discriminator is fed output of the decoder branch fine pointcloud and the output is either 1 for synthetic dataset element or 0 for real dataset element. Therefore, as loss function, we are able to use binary cross entropy; see Equation (7) below. Discriminator is trained until $\epsilon_{\Phi 3}<0.05$, then the final training phase can begin.

(7) $$\epsilon_{\Phi 3}=BCE\left(y,\hat{y}\right)=\sum_{i=1}^{N}\hat{y_{i}}\cdot log\left(y_{i}\right)+\left(1-\hat{y_{i}}\right)\cdot log\left(1-y_{i}\right).$$

#### 3.3.4. Phase IV

During the fourth and final phase, the actual adversarial training is performed for pointcloud refinement. Because we want our training to start afresh, we drop all previous optimizer states. This helps to kick-start the training in case optimizers have built up local minima states. During the adversarial training phase, we train on both synthetic and real datasets. Synthetic dataset is used to further enforce the decoder state and to reinforce discriminator, while real data is used to update the refiner/encoder weights and to reinforce discriminator. Phase IV consists of three different steps for each batch that is being trained with the weights being updated separately. The first step of Phase IV reinforces the entire network using the synthetic dataset, for loss function for step one see Equation (8). The second step of the phase involves training refiner using adversarial loss, where the network attempts to predict such a pointcloud for real world data that the discriminator would be fooled into thinking that this is a synthetic data sample. During this step, only the refiner weights are being updated; the discriminator is left untouched, and only its loss function is used (see Equation (9)). In addition, we do not want the refiner to lose the shape of the point cloud, and, for this reason, we constrain it with pointcloud loss in relation to the input frame with a factor of α=0.4, the value of which was chosen experimentally. This allows the pointcloud to gain adversarial properties and actually refine the model without losing the underlying shape. And, finally, we reinforce the discriminator to recognize the real dataset pointclouds from synthetic (see Equation (10)).

(8) $$\epsilon_{\Phi 4a}=EMD\left(S_{clean},{\hat{S}}_{clean}\right)+\epsilon_{\Phi 2}+\epsilon_{\Phi 3},$$

(9) $$\epsilon_{\Phi 4b}=\alpha \cdot EMD\left(S_{clean},{\hat{S}}_{clean}\right)+\gamma \cdot EXP\left(S_{clean},{\hat{S}}_{fine}\right)+BCE\left(1-y,\hat{y}\right),$$

(10) $$\epsilon_{\Phi 4c}=\epsilon_{\Phi 3}.$$

## 4. Results

**Figure 11:**
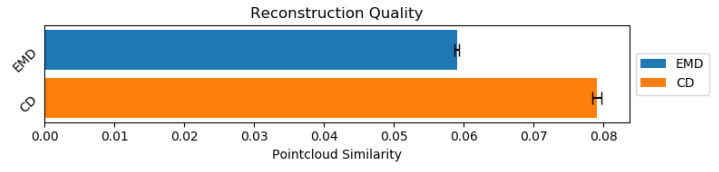
Reconstruction similarity using both *Earth Movers Distance* and *Chamfer Distance*. Lower values are better.

The main purpose of our approach is to reconstruct self-occluded human body shapes using real world depth sensor data. However, it is not possible to objectively measure the achieved results for real world data as we have no way of comparing ground truth with prediction, no such ground truth pointclouds exist. For this reason, we will only objectively measure the reconstruction quality using synthetic dataset, while the evaluation of the real world data will be evaluated using expert knowledge. For evaluating synthetic dataset, we will use two quality metrics *Chamfer* (see Equation (11)) and *Earth Mover’s* distance. Results for synthetic dataset reconstruction are shown in Figure 11.

(11) $$\epsilon_{cd}\left(S,\hat{S}\right)=\frac{1}{2}\left(\frac{1}{\left|S\right|}\sum_{x\in S}\underset{y\in \hat{S}}{min}||x-{y||}_{2}^{2}+\frac{1}{\hat{S}}\sum_{y\in \hat{S}}{\underset{x\in S}{min}||x-y||}_{2}^{2}\right).$$

To compare our approach to other state-of-the-art research using both *Earth Mover’s* and *Chamfer* distance metrics, from Table 2, we can see that the other approaches, without modifications, completely fail when attempting to deal with our *AMASS* dataset, despite having comparable results (Liu et al. [38]) when applied *ShapeNet* dataset to our results with *AMASS* dataset. Unfortunately, we cannot compare our approach against *ShapeNet* dataset as our suggested approach is meant for the synthesis of real-world object data for reconstruction. Comparing against it would require collection of a real world *ShapeNet*-like dataset using depth sensors, in addition to generation of synthetic frames.

**Figure 12:**
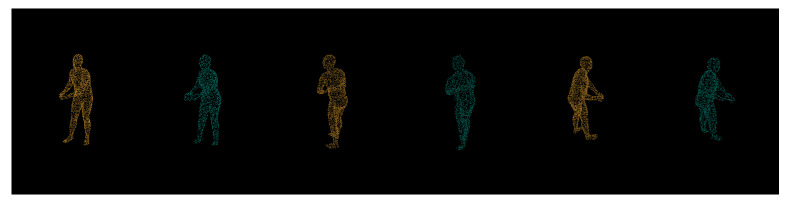
Comparison of ground truth (left/orange) and prediction (right/teal) from different viewpoints.

Additionally, we inspect synthetic and real world data results visually using expert knowledge. Figure 12 depicts synthetic models input (orange) being compared side by side. As we can see, the majority of the reconstruction flaws occur at the ends of the limbs (both hands and feet) due to those features requiring much finer granularity. Alongside ground-truth to prediction side by side comparisons, we perform input-to-prediction overlap visual inspection as it helps us see to better compartmentalize what features were given as the input to neural network and what it had to make a guess.

**Figure 13:**
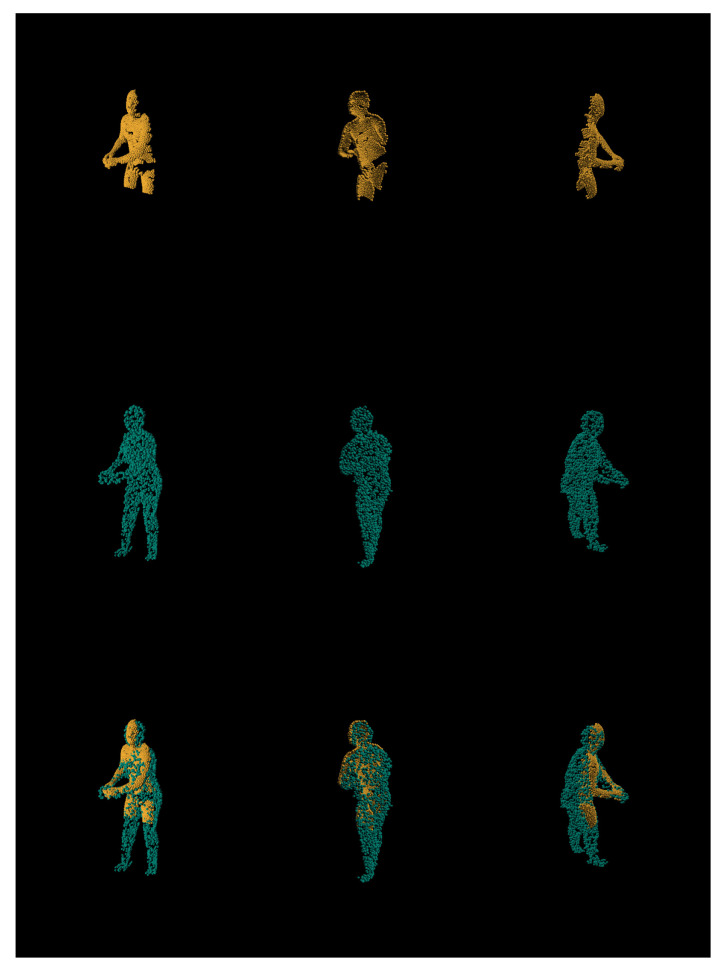
Stacked views for synthetic input (orange) and its prediction (teal).

**Figure 14:**
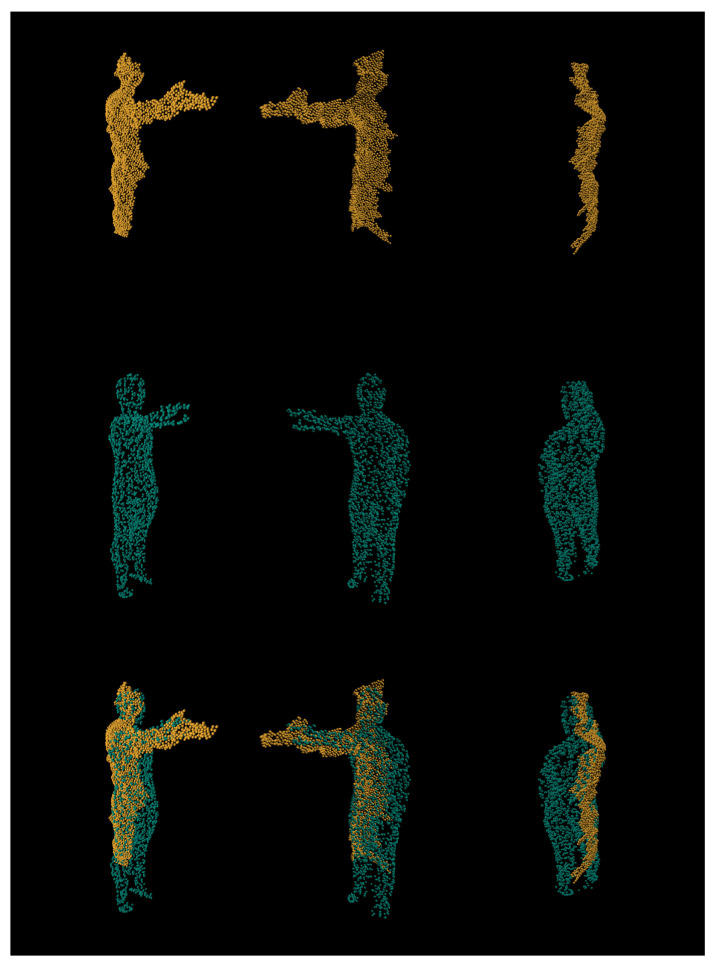
Stacked views for real depth sensor input (orange) and its prediction (teal). Real input is quite distorted due to depth sensor inaccuracies, and the cleanup stage manages to clean up the majority of the noise.

From Figure 13, we can see that, despite being given very little input (orange) about legs, our network has managed to predict (teal) the entirety of its orientation. As we can see, the network has managed to predict and reconstruct the entire human posture, while being given less than half of the body features. Finally, we compare real world data reconstruct in comparison to the input depth (see Figure 14).

As we can see, the network has had no issue in reconstructing the human posture and cleaning out the majority of deformations. The biggest defects for real world data reconstruction are where the depth has large deformities; for example, in the top row, we can see that, at the end of the hand, there is an extra lump that was captured by the depth sensor. This has caused the reconstruction prediction to fail cut off part of the hand. Additionally, there visually seem to be small scaling discrepancies between input and reconstruction, although these discrepancies are somewhat hard to evaluate, even with expert knowledge; regardless of that, we can safely assert that the addition of adversarial refinement to the network has allowed the network to understand and reconstruct real world depth sensor data, whereas other approaches have failed without. Finally, we have tried applying our approach to *ITOP* dataset. However, due to very different noise model and point distribution of *Microsoft Kinect* sensor when compared to *Intel Realsense*, the reconstruction results proved to be inconsistent. Having the model trained with *ITOP* dataset would greatly improve the results. Unfortunately, the dataset has additional background noise that we were unable to isolate to be used during the training process. Further research could improve our model by making it compatible with *Kinect*-like device noise; however, due to the *Microsoft Kinect* being a discontinued product, we feel like pursuing this is not as useful.

## 5. Discussion

The main of advantage over other state-of-the-art approaches involving object reconstruction, is that our three-staged neural network architecture is capable of reconstructing full human body postures with no external interference using real world depth sensor frames. The addition of the cleanup stage has allowed our approach to not only denoise the input data, but it also acted as real world input refinement, which has allowed us to train the network without actually having ground truths for it. Furthermore, our solution, unlike voxel grid-based approaches, does not require us finding the objects’ transformation matrix in order to scale and translate it to place it in 3D space. Instead, it is easily adaptable to existing 3D applications, such as AR or VR. Finally, while our solution had no issue in reconstructing the general objects’ shape with less than half of the object visible, finer details, like palms and fingers, tend to be too small features for reconstruction, causing ambiguities and bad reconstruction results.

## 6. Conclusions

We proposed three-staged adversarial auto-refining reconstruction network, which is capable of reconstructing human body using both: synthetic depth inputs and real world depth sensor data. The network has achieved *Earth Mover’s* and *Chamfer* distances of 0.059 and 0.079, respectively, indicating good reconstruction quality, when compared to other state-of-the-art methods. Additionally, when comparing the reconstructed pointclouds visually, it is clear that the network manages to meet the expectations of reconstructing both synthetic and real world samples with most of the defects being concentrated at the ends of the limbs, or in the case of real world data, large defects in the depth map can cause defects in the reconstruction even when cleaning. This is likely due to constraints of refiner attempting to retain the original shape of the object.

Finally, we have proposed a four-phased training approach for training the adversarial auto-refiner. The addition of adversarial refinement to the network has allowed our approach to work with real-world depth sensor data, which other approaches are unable to do.

## Figures and Tables

**Table 1:** Comparison between different implementations. Networks capable of performing sensor-to-screen prediction where no additional external help is required are marked as Standalone.

Name	Voxels	Pointcloud	Input	EMD	CD	Standalone
3D-R2N2	✓	✗	RGB	—	—	✗
YoloExt	✓	✗	RGB-D	—	—	✓
PointOutNet	✗	✓	RGB	✓	✓	✗
PointNet w FCAE	✗	✓	Pointcloud	✗	✓	✗
PCN	✗	✓	Pointcloud	✗	✓	✗
AtlasNet	✗	✓	Pointcloud	✗	✓	✗
MSN	✗	✓	Pointcloud	✓	✗	✗
Ours	✗	✓	Depth	✓	✗	✓

**Table 2:** Comparison of different reconstruction method metrics for different reconstruction methods and ours. Our approach when applied to *AMASS* dataset has very similar metrics to state-of-the-art approaches on *ShapeNet* datasets, whereas other methods completely fail when reconstructing *AMASS* dataset. Our method is not applicable to *ShapeNet* as our data collection and training process is more complicated.

Method	ShapeNet		AMASS	
	EMD	CD	EMD	CD
PointNet wFCAE [21]	0.0832	0.0182	3.3806	4.9042
PCN [36]	0.0734	0.0121	3.0456	4.0955
AtlasNet [37]	0.0653	0.0182	2.0875	6.4343
MSN [38]	0.0378	0.0114	1.1525	0.8016
Our method (Cleanup)	*N/A*	*N/A*	0.0603	0.0292
Our method (Reconstruction)	*N/A*	*N/A*	0.0590	0.0790

## Data Availability

The data is available from the corresponding author upon reasonable request.
